# The endogenous soluble VEGF receptor-2 isoform suppresses lymph node metastasis in a mouse immunocompetent mammary cancer model

**DOI:** 10.1186/1741-7015-8-69

**Published:** 2010-11-03

**Authors:** Masa-Aki Shibata, Jayakrishna Ambati, Eiko Shibata, Romulo JC Albuquerque, Junji Morimoto, Yuko Ito, Yoshinori Otsuki

**Affiliations:** 1 Department of Anatomy and Cell Biology, Division of Life Sciences, Osaka Medical College, Osaka, Japan; 2 Department of Ophthalmology and Visual Sciences and Physiology, University of Kentucky, Lexington, KY, USA; 3 Laboratory for Drug Discovery Innovation, Department of Molecular Pharmacology, National Cerebral & Cardiovascular Center Research Institute, Osaka, Japan; 4 Laboratory Animal Center, Osaka Medical College, Osaka, Japan

## Abstract

**Background:**

Cancer metastasis contributes significantly to cancer mortality and is facilitated by lymphangiogenesis and angiogenesis. A new splicing variant, endogenous soluble vascular endothelial growth factor receptor-2 (esVEGFR-2) that we recently identified is an endogenous selective inhibitor of lymphangiogenesis. To evaluate the antimetastatic potential of esVEGFR-2, gene therapy with vector expressing esVEGFR-2 (pesVEGFR-2) or endostatin (pEndo) as a positive control was conducted on murine metastatic mammary cancer.

**Methods:**

Syngeneic inoculated metastatic mammary cancers received direct intratumoral injection of pesVEGFR-2, pEndo or pVec as control, once a week for six weeks. *In vivo *gene electrotransfer was performed on the tumors after each injection.

**Results:**

Deaths from metastasis were much lower in the pesVEGFR-2 and pEndo groups than in those of the pVec. Tumor volume was significantly lower in the pesVEGFR-2 and the pEndo groups throughout the study. Multiplicity of lymph node and lung metastatic nodules was significantly suppressed in the pesVEGFR-2 and pEndo groups. Moreover, the total number of overall metastasis including the other organs was also decreased in these groups. However, pesVEGFR-2 was not able to decrease the number of lungs, ovaries, kidneys and adrenals with metastasis as counted by unilateral or bilateral metastasis. The number of CD34^+^/Lyve-1^- ^blood microvessels was significantly decreased in the pEndo group, while the number of CD34^-^/Lyve-1^+ ^lymphatic vessels was significantly decreased in the pesVEGFR-2 and pEndo groups. In addition, a significant reduction in the number of dilated lymphatic vessels containing intraluminal cancer cells was observed in the pesVEGFR-2 and pEndo groups. Levels of apoptosis were significantly increased in the pEndo group, whereas the rates of cell proliferation were significantly decreased in the pesVEGFR-2 and pEndo groups.

**Conclusions:**

Our data demonstrate that esVEGFR-2 can inhibit mainly lymph node metastasis. The antimetastatic activity of esVEGFR-2 may be of high clinical significance in the treatment of metastatic breast cancer because lymph node involvement is a most important prognostic factor in cancer patients.

## Background

The majority of cancer deaths are due to metastatic spread of tumor cells. The mortality rate among breast cancer patients is also largely the result of metastasis, the common sites being the lymph nodes, lung, liver and bone. Lymph node metastasis is one of the most important adverse prognostic factors for breast cancer [1]. In principle, cancer cells spread through the body by different mechanisms, such as direct invasion of surrounding tissue, hematogenous metastasis and/or lymphatic metastasis. Thus, development of vascular supply is a key factor in the growth and metastatic spread of cancers. The ability to block the signaling system that enables the spread of cancer would be a major step forward in the prevention of tumor metastasis, and would consequently reduce both morbidity and mortality.

The vascular endothelial growth factor (VEGF) family of molecules is critical for vascular development and pathological sprouting. The growth of blood vessels (angiogenesis) is primarily initiated by activation of VEGFR-1 and VEGFR-2 by VEGF-A, whereas lymphangiogenesis is predominantly driven by VEGF-C, which activates VEGFR-2 and VEGFR-3 expressed in lymphatic endothelial cells. Recently, blockade of VEGFR-3 signaling by soluble VEGFR-3 (sVEGFR-3) or the blocking antibody inhibits lymph node metastasis in experimental animal cancer models and associated with reduction in lymphangiogenesis but not anginogenesis of the tumors [2-6]. More recently, an endogenous soluble isoform of VEGFR-2 (esVEGFR-2) that sequesters VEGF-C was identified and shown to be the first endogenous specific inhibitor of lymphatic vessel growth [7]. esVEGFR-2 is a truncated form of 230 kDa membrane-bound form of VEGFR-2 resulting from alternative splicing. In addition, tissue-specific loss of esVEGFR-2 in mice induces, at birth, spontaneous lymphatic invasion of the normally alymphatic cornea and hyperplasia of skin lymphatics without affecting angiogenesis. Treatment with esVEGFR-2 inhibits lymphangiogenesis but not angiogenesis induced by corneal suture injury or transplantation, enhances corneal allograft survival and suppressed lymphangioma cell proliferation [7].

VEGF-C is the major lymphangiogenic factor highly expressed in a variety of malignant tumors including mammary cancer [8]. Furthermore, over-expression of VEGF-C has been reported to be associated with a poor prognosis and lymph node metastasis in breast cancer patients [9,10]. A number of animal studies using cell lines [2,11,12] and transgenic mice [13] have been conducted in an attempt to demonstrate that VEGF-C over-expression is able to promote cancer metastasis. Thus, tumor cell-derived VEGF-C is thought to enhance lymph node metastasis. Moreover, VEGF-A is well-known to exert a crucial role in tumor angiogenesis [14]. An adequate blood supply is required to sustain the uncontrolled cell proliferation characteristic of malignant tumors, and tumorigenesis and metastasis have been associated with angiogenesis in tumors [14]. Therefore, lymphangiogenesis and angiogenesis in tumors have become potential targets for cancer therapy. The recent discovery of esVEGFR-2 [7] and its selective inhibition of VEGF-C signaling, led to the interrogation of whether it would serve as a therapeutic tool for preventing cancer metastasis and dissecting the precise individual contribution of lymphangiogenesis and VEGF-C signaling in this milieu.

In the present study, we examined whether gene therapy with an alternative splicing variant esVEGFR-2 (an endogenous inhibitor of lympphangiogenesis) might lead to suppression of lymphatic metastasis in a mouse immunocompetent mammary cancer model. In addition, since endostatin is also a naturally occurring molecule and exerts both inhibitions of blood and lymphatic vessels, this protein served as a positive control [15,16].

## Methods

### Vectors

The open reading frame of e*sVegfr2 *was cloned from mouse corneal cDNA and inserted into a pcDNA3.1 vector for *in vivo *overexpression as previously described [7]. Empty vector pcDNA3.1 was used as a control vector and referred to as pVec. The plasmid pBLAST-mEndo XVIII (InvivoGen, Inc. San Diego, CA, USA), which encodes murine endostatin with the addition of the IL-2 signal sequence for secretion, was used as a positive control [15]. For simplicity in this manuscript, the vectors are referred to as pesVEGFR-2 and pEndo, respectively. All plasmid vectors were extracted from *Escherichia coli *(DH5α strain) and purified by means of a modified alkaline lysis procedure using a Plasmid Maxi Kit (Qiagen Inc., Valencia, CA, USA) and further purified with centrifugal filters (Ultrafree-MC, Millipore Co., Bedford, MA, USA).

### Cell line and animals

Mouse mammary tumor virus (MMTV), purified from medium in which Jyg-MC cells (established from mammary tumors of the Chinese wild mouse) were grown, was inoculated into the inguinal mammary glands of female BALB/c mice, resulting in the development of mammary carcinomas [17]. The BJMC3879 mammary adenocarcinoma cell line was subsequently derived from a metastatic focus within a lymph node from one of the inoculated BALB/c mice and the cell line continues to show a high metastatic propensity, especially to lymph nodes and lungs, a trait retained through culture [18-20]. This cell line and inoculated tumors expressed VEGF-C and VEGFR-3 [21]. The BJMC3879luc2 mammary carcinoma cell line was generated by stable transfection with *luc2 *gene (an improved *firefly luciferase *gene) to parent cell line BJMC3879 cells [22]. BJMC3879luc2 cells were maintained in RPMI 1640 medium containing 10% fetal bovine serum with streptomycin/penicillin in an incubator under 5% CO_2 _at 37°C.

A total of 30 female, six-week-old BALB/c mice was used in this study (Japan SLC, Hamamatsu, Japan). The animals were housed five per plastic cage on wood chip bedding with free access to water and food under controlled temperature (21 ± 2°C), humidity (50 ± 10%), and lighting (12:12 hour light:dark cycle). All animals were held for a one-week acclimatization period before study commencement. All manipulations of mice were performed in accordance with the procedures outlined in the Guide for the Care and Use of Laboratory Animals in Osaka Medical College, Japanese Government Animal Protection and Management Law (No.105) and Japanese Government Notification on Feeding and Safekeeping of Animals (No.6).

### *In vivo *esVEGFR-2 gene therapy on mammary cancer model

BJMC3879luc2 cells (2.5 × 10^6 ^cells/0.3 ml in phosphate buffered saline) were inoculated into the right inguinal region of 30 female BALB/c mice. The animals were then randomly allocated into three groups of 10 mice each: the pVec (control), pesVEGFR-2, or pEndo groups. Two weeks post-inoculation, when the tumors had reached 0.4 to 0.5 cm in diameter, we injected pVec, pesVEGFR-2 or pEndo directly into the tumors and then immediately performed *in vivo *gene electrotransfer by applying a conductive gel (Echo Jelly; Aloka Co., Ltd., Tokyo, Japan) topically to the unshaved skin over the injected tumor. The vectors were injected using a 27-gauge needle at a concentration of 0.5 μg/μl in sterile saline while the animals were under isoflurane anesthesia. A total volume of 150 μl was introduced into larger tumors, while smaller tumors of 0.6 to 0.7 cm were infused until we detected leakage of the vector solution. Electric pulses were delivered directly to the tumor via "forceps" platinum plate electrodes (CUY650-10; Nepa Gene Co. Ltd., Ichikawa, Japan) using a CUY21EDIT square-wave electropulser (Nepa Gene Co., Ltd.). Conditions for gene electrotransfer used in the present study were intratumoral injection of 50 to 75 μg plasmid (dependent on tumor size as mentioned above), eight pulses with a pulse length of 20 milliseconds at 100 volts. The parameters for optimal gene electrotransfer were previously determined [15,18,20].

Using calipers, we measured the size of each treated mammary tumor weekly and calculated tumor volumes using the formula *maximum diameter *× *(minimum diameter)*^*2 *^× *0.4 *[23]. Individual body weights were also recorded at weekly intervals. All surviving animals were injected intraperitoneally with 50 mg/kg 5-bromo-2'-deoxyuridine (BrdU; Sigma Co., St. Louis, MO, USA) at one hour prior to sacrifice. After six weeks of treatment, all mice were euthanized under isoflurane anesthesia and the mammary tumors and certain lymph nodes (that is, nodes from axillary and femoral regions as well as any that appeared abnormal) were removed. We then immediately fixed a portion of each tissue sample in 10% phosphate-buffered formalin and processed through to paraffin embedding; an additional portion of each tumor was also immediately frozen in liquid nitrogen for molecular analysis. Lungs were routinely inflated with the fixative, excised and immersed back into the fixative. We subsequently trimmed and examined all lobes for metastatic foci before processing through histology, where they were cut into 4-μm-thick sections and stained with hematoxylin and eosin (H&E) for histopathological examination or remained unstained sections for immunohistochemistry.

### Bioluminescence imaging *in vivo*

At Week 6, while under isoflurane inhalation using an SBH Scientific anesthesia system (SBH Designs Inc., Windsor, Ontario, Canada), a minimum of five mice from each group were injected intraperitoneally with D-luciferin potassium salts (Wako Pure Chemical Industries, Osaka, Japan) at 3 mg/mouse. Bioluminescence imaging with a Photon Imager (Biospace Lab, Paris, France) was performed. The bioluminescent signals received during the six-minute acquisition time were imaged and quantified using Photovision software (Biospace Lab).

### p53 immunohistochemistry

The labeled streptavidin-biotin (LSAB) method (Dako Co., Glostrup, Denmark) was used for p53 immunohistochemistry. Unstained sections were immersed in distilled water and heated for antigen retrieval prior to incubation with a p53 mouse monoclonal antibody (Clone Pab240; Santa Cruz Biotechnology, Santa Cruz, CA, USA) that reacts to the mutant protein in fixed specimens.

### Blood and lymphatic microvascular densities in mammary tumors

Immunohistochemistry was performed on samples using the blood and lymphatic vessel markers CD34 and Lyve-1 respectively to quantitatively assess the number of microvessels present in primary mammary carcinomas. Rat anti-CD34 (Hycult Biotech, Uden, The Netherlands) and rabbit anti-LYVE-1 (Acris Antibodies GmbH, Herford, Germany) were used as primary antibodies and were detected using goat anti-rat Alexa-594 and goat anti-rabbit Alexa-488 (Molecular Probes, Life Technol. Corp., Carlsbad, CA, USA). Nuclear staining was performed with Vectashield mounting medium with DAPI (Vector Labs, Inc., Burlingame, CA, USA). The probes were then visualized at high magnification (x200) using a laboratory microscope equipped with a high pressure mercury burner for fluorescence (Olympus Co., Tokyo, Japan). The mammary carcinoma tissues immunohistochemically stained were observed and digitally captured whole periphery of the tumors at high magnification (x200) under fluorescence with a 590 nm or 495 nm excitation filter. The corresponding three images (CD34, Lyve-1 and DAPI) were merged into a single image and the number of CD34^+^/Lyve-1^- ^and the number of CD34^-^/Lyve-1^+ ^vessels were counted.

### Dilated lymphatic vessels with cancer cell invasion

Mammary tumor sections from paraffin-embedded tissues were immunohistochemically stained using the LSAB method (Dako Co.). A hamster anti-podoplanin monoclonal antibody (AngioBio Co., Del Mar, CA, USA), against a lymphatic endothelium marker was used. To quantitatively assess the number of lymphatic vessels having intraluminal tumor cells in whole periphery area of the primary mammary carcinomas, the slides were scanned at low-power (x100) magnification to identify podoplanin-positive lymphatic vessels, and were then confirmed whether the lymphatic vessel contain mammary cancer cells or not at higher (x200 to 400) magnification. The number of podoplanin-positive lymphatic vessels containing intraluminal tumor cells in whole periphery of the tumors was counted and expressed as the average ± SD.

### Apoptosis and cell proliferation in mammary tumors

For the quantitative analyses of apoptotic cell death, sections from paraffin-embedded tumors were assayed using the terminal deoxynucleotidyl transferase-mediated dUTP-FITC nick end-labeling (TUNEL) method in conjunction with an apoptosis *in situ *detection kit (Wako Pure Chemical Industries) with minor modifications to the manufacturer's protocol. TUNEL-positive cells (mainly regarded as apoptotic cells) were counted in viable regions peripheral to areas of necrosis in tumor sections. The slides were scanned at low-power (x100) magnification to identify those areas having the highest number of TUNEL-positive cells; four areas neighboring the highest area of TUNEL-positive cells were then selected and counted at higher (x200 to 400) magnification to obtain mean ± SD values. The numbers of TUNEL-positive cells were expressed as numbers per cm^2^.

The tumors from five animals from each treatment group were subsequently evaluated for cell proliferation rates (BrdU labeling indices) as inferred by BrdU incorporation. DNA was denatured *in situ *by incubating unstained paraffin-embedded tissue sections in 4 N HCl solution for 20 minutes at 37°C. The incorporated BrdU was visualized after exposure to an anti-BrdU mouse monoclonal antibody (Clone Bu20a, Dako Co.). The numbers of BrdU-positive S-phase cells per 250 mm^2 ^were counted in four random high power (×200) fields of viable tissue, and the BrdU labeling indices were expressed as numbers per cm^2^.

### Statistical analyses

Significant differences in the quantitative data between groups were analyzed using the Student's *t*-test via the Welch method which provides for insufficient homogeneity of variance. The differences in metastatic incidence were examined by Fisher's exact probability test, with *P *< 0.05 or *P *< 0.01 considered to represent a statistically significant difference.

## Results

### Survival rates, body weights and tumor growth in gene therapy using pesVEGFR-2 or pEndo

**Figure 1:**
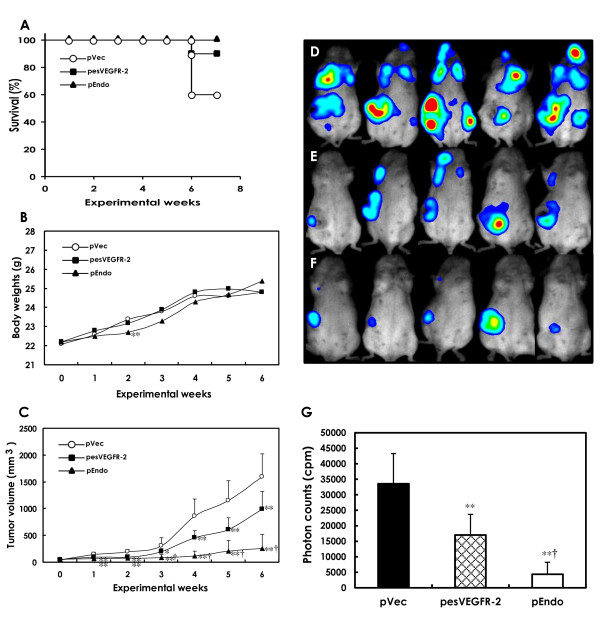
**Survival rates, body weights, tumor volumes and bioluminescent imaging in mice receiving esVEGFR-2 gene therapy**. Survival rates (**A**), body weights (**B**) and tumor volumes (**C**) in mammary carcinomas from female BALB/c mice transfected with pVec (control), pesVEGFR-2 and pEndo vectors using *in vivo *gene electrotransfer. Each group consisted of 10 mice. **A**. Survival rates were much lower in the pVec group than in those of the pesVEGFR-2 and pEndo groups. **B**. Body weights were similar between pVec group and pesVEGFR-2 or pEndo groups with exception of the pEndo group at Week 2. **C**. From Week 1 on, rates of tumor growth (tumor volumes) in the pesVEGFR-2 and pEndo groups were significantly decreased as compared to the control values, the differences becoming even more pronounced by the termination of the experiment (Week 6) (**P < 0.01 as compared to the pVec group; †*P *< 0.01 as compared to the pesVEGFR-2 group). Data presented are means ± SD values. **D-F**. Bioluminescent imaging of five mice representative of each group. Bioluminescent imaging showed a tendency for decreases in extension of metastasis in the pesVEGFR-2 (**E**) and pEndo (**F**) groups as compared to the pVec group (**D**). **G**. Quantification of the average bioluminescent signals was significantly decreased in those of the pesVEGFR-2 and pEndo groups (**P < 0.01) as compared to the control values. As compared to the pesVEGFR-2 group, the levels were further decreased in the pEndo group (†*P *< 0.01).

Survival rates are shown in Figure 1A. Four animals (40%) in the pVec (control) group and one animal (10%) in the pesVEGFR-2 group died at Week 6 due to the widespread metastasis of mammary carcinoma. Reduction in body weight of mice was not seen as a result of any treatment regimen with the exception of the pEnd group at Week 2 but recovered thereafter (Figure 1B). The general condition of the animals was good throughout the experiment. Data for tumor volumes are presented in Figure 1C. Tumor volume increases were significantly suppressed in the pesVEGFR-2 and pEndo groups from Week 2 to the end of the study as compared to the pVec group. Tumor volumes in the pEndo group (positive control) were significantly smaller than those in the pesVEGFR-2 group from Week 3 to the end of the study (Figure 1C).

### Mammary carcinoma metastasis

#### Bioluminescence imaging

Metastasis was visualized by bioluminescence imaging at Week 6 (Figure 1D-F). The imaging showed a tendency for decrease of metastatic expansion in mice treated with pesVEGFR-2 (Figure 1E) and pEndo (Figure 1F) as compared to control animals (Figure 1D). The magnitude of metastasis was measured in each group by bioluminescence imaging. As shown in Figure 1G, the levels were significantly decreased in the pesVEGFR-2 and pEndo groups as compared to the control group. In addition, as compared to the pesVEGFR-2 levels, the levels in the pEndo group were significantly decreased.

#### Primary implanted mammary carcinoma

**Figure 2:**
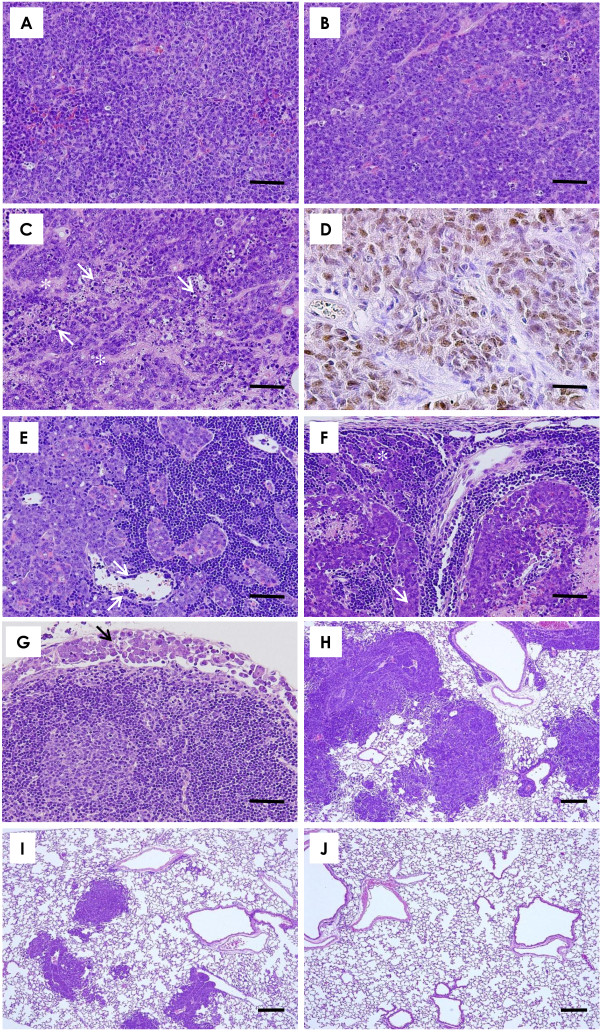
**Histopathological findings in mice receiving esVEGFR-2 gene therapy**. The implanted mammary carcinomas proved to be moderately differentiated adenocarcinoma (**A-C**, scale bar = 50 μm). Histopathologically, no apparent differences in mammary carcinomas were found between the pVec (**A**) and pesVEGFR-2 (**B**) groups. However, mammary carcinomas in the pEndo group showed spoke-like cell death regions (asterisks) within viable tumor cells (**C**). The cell death region was observed around blood vessels (asterisk) and apoptotic bodies (arrows) were seen along with the cell death area (**C**). p53 immunohistochemistry of mammary carcinoma induced by BJMC3879luc2 cell inoculation (**D**, scale bar = 25 μm). Note nuclear staining for abnormal p53 protein, indicating that these cells carry mutant p53. Metastasis to lymph node in the pVec (**E**), pesVEGFR-2 (**F**) and pEndo (**G**) (**E-G**, scale bar = 50 μm). Metastatic carcinoma cells were presented in medullary cord and intraluminal space of the blood vessel (**E**, arrows). Metastatic carcinoma cells were filled with subcapsular sinus (asterisk) and cortical sinus (arrow) (**F**). Fewer lymph nodes with metastasis were found in the therapeutic groups. Metastatic tumor cells were observed in subcapsular sinus (**G**, arrow). Metastatic foci in the lung of the pVec, pesVEGFR-2 and pEndo groups (**H-J**, scale bar = 200 μm). Many metastatic foci and nodules with small to large were seen in the pVec group (**H**). Metastatic lung foci were much smaller in the pesVEGFR-2 (**I**) group than in the control group (J). No metastatic foci were observed in the lung of mouse given pEndo (**J**). **A-C **and **E-J**, H&E stain; **D**, p53 immunohistochemistry.

Histopathologically, the mammary carcinomas induced by BJMC3879luc2 cell inoculation proved to be moderately differentiated adenocarcinomas (Figure 2A), which contain p53 mutation as inferred by immunohistochemistry (Figure 2D). The mammary carcinomas showed no apparent differences between the pVec (Figure 2A) and pesVEGFR-2 (Figure 2B) groups. However, the mammary carcinoma transfected with pEndo showed spoke-like cell death regions within viable tumor tissue (Figure 2C). The cell death region was observed along with blood microvessels and apoptotic bodies were also seen in association with the cell death areas (Figure 2C).

#### Metastasis to lymph nodes

**Figure 3:**
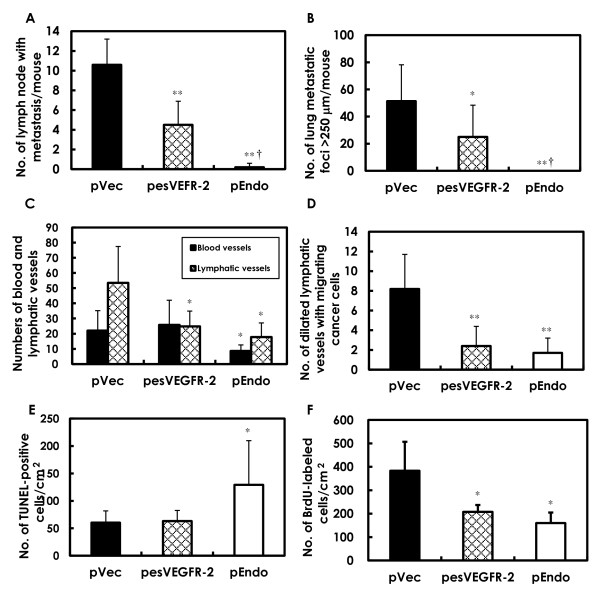
**Quantitative analyses of metastasis, vascular density, apoptosis, cell proliferation in mammary carcinomas**. **A**. Multiplicity of lymph node metastasis was significantly decreased in the pesVEGFR-2 and pEndo groups (**P < 0.01 as compared to the pVec group; †*P *< 0.01 as compared to the pesVEGFR-2 group). **B**. Multiplicity of lung metastatic foci > 250 μm was significantly reduced in the pesVEGFR-2 and pEndo groups (**P < 0.01 as compared to the pVec group; †*P *< 0.01 as compared to the pesVEGFR-2 group). **C**. Quantitation of blood and lymphatic microvessels were conducted using CD34 and Lyve-1 immunohistochemistry. The number of CD34^+^/Lyve-1^- ^blood vessels was significantly decreased in the pEndo group, but not in the pesVEGFR-2 group, as compared to the pVec group. The number of CD34^-^/Lyve-1^+ ^lymphatic microvessels was significantly decreased in the pesVEGFR-2 and pEndo groups. **D**. The mammary tumors were immunohistochemically stained for another lymphatic endothelial marker podoplanin. The number of dilated lymphatic microvessels containing intraluminal tumor cells was significantly lower in pesVEGFR-2 and pEndo groups than those observed in the control pVec group. **E**. Apoptotic cell death, assessed by TUNEL assay, was significantly increased in the pEndo group. **F**. Cell proliferation, inferred by BrdU labeling indices, was significantly decreased in the pesVEGFR-2 and pEndo groups. *P < 0.05 and **P < 0.01 as compared to the values of the pVec group. Data presented are means ± SD values.

Representative lymph node metastases are presented in Figure 2E-G. Many metastatic tumor cells were found in lymphatic sinus (Figure 2E-G). As shown in Figure 3A, the number of lymph node metastases per mouse was significantly lower in the pesVEGFR-2 and the pEndo groups as compared to the pVec group. Inhibition of lymph node metastasis in the pEndo group (positive control) was significantly stronger than in those observed in the pesVEGFR-2 group (Figure 3A).

#### Metastasis to lungs

Histopathologically, large metastatic lung nodules tended to be few in the pesVEGFR-2 group (Figure 2I) as compared to the pVec group (Figure 2H). In the pEndo group, no metastatic foci were found (Figure 2J). Quantitative measurements of the numbers of metastatic lung nodules > 250 μm per mouse showed a significant inhibition of metastasis upon exposure to pesVEGFR-2 or pEndo (Figure 3B). The number of metastatic nodules in the pEndo group was significantly decreased as compared to the pesVEGFR-2 group (Figure 3B).

#### Overall metastasis

In metastasis to other organs, metastatic foci were observed in ovaries, kidneys, adrenals and uterus. With respect to bilateral organs, one in unilateral metastasis and two in bilateral metastasis were counted. The multiplicity of overall metastasis is presented in Table 1. Total numbers of organs with metastasis tended to be much lower in the pesVEGFR-2 and pEndo groups as compared to the pVec group. The average number of all organs with metastasis was significantly decreased in the pesVEGFR-2 and pEndo groups as compared to the pVec group. In addition, as compared with the pesVEGFR-2 group, the pEndo group (positive control) showed further significant suppression of the average number of organs with metastasis (Table 1). Furthermore, as shown in the average number of individual organs with metastasis as counted by unilateral or bilateral metastasis, pesVEGFR-2 did not inhibit metastasis to lungs, ovaries, kidneys and adrenals (Table 1). In contrast, pEndo significantly decreased in all organs with metastasis as compared to the control or pesVEGFR-2 groups.

**Table 1:** Multiplicity of any category of metastasis^a)^

				Average No. of organs with metastasis except **for lymph nodes**^**b)**^				
**Groups**	**Number of mice examined**	**Total no. of organs with metastasis**	**Average no. of organs with metastasis/mouse^b)^**	**Lungs**	**Ovaries**	**Kidneys**	**Adrenals**	**Others**

pVec	9	156	16.3 ± 3.3	2.0 ± 0.0	1.9 ± 0.3	1.6 ± 0.5	1.3 ± 0.9	0.0 ± 0.0

pesVEGFR-2	10	106	9.9 ± 4.1**	1.5 ± 0.8	1.8 ± 0.6	1.1 ± 1.0	1.6 ± 0.8	0.1 ± 0.3^c)^

pEndo	9	6	0.7 ± 1.1**†	0.0 ± 0.0**†	0.0 ± 0.0 **†	0.0 ± 0.0**†	0.3 ± 0.7 *†	0.1 ±0.3^d)^

*P < 0.05; **P < 0.01 as compared with pVec values.

†*P *< 0.01 as compared with pesVEGFR-2 values.

a) With respect to bilateral organs, one in unilateral metastasis and two in bilateral metastasis were counted.

b) Values are mean ± SD.

c) Metastasis to uterus.

d) Metastasis to subcutis.

### Blood and lymphatic microvessels in treated mammary tumors

**Figure 4:**
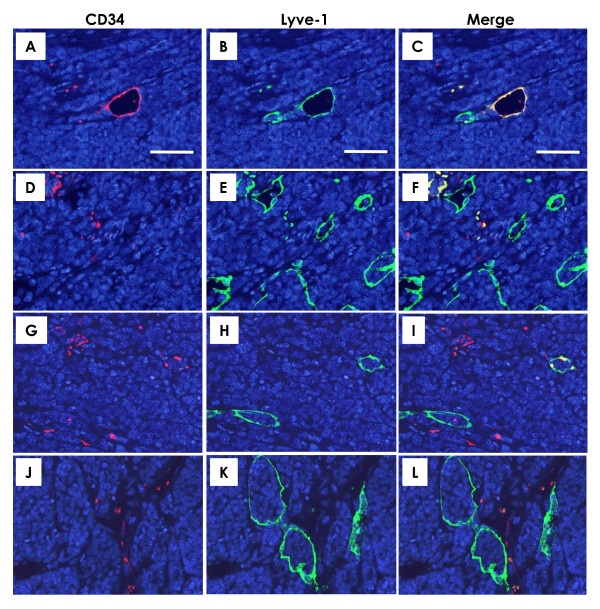
**Immunofluorescence for blood and lymphatic vessels in mammary carcinomas**. Double immunohistochemical staining with CD34 for blood microvessels (red) and Lyve-1 for lymphatic (green) microvessels and their merged images in mammary tumors. As can be seen in their merged image (**C**), some microvessels showed both expressions of CD34 (**A**) and Lyve-1 (**B**). Such microvessels having CD34^+^/Lyve-1^+ ^characteristics were excluded from quantitation. The number of lymphatic microvessels was lower in the pesVEGFR-2 (**G-I**, merge in **I**) and pEndo (**J-L**, merge in **L**) groups than in the pVec group (**D-F**, merge in **F**). **A**-**L**, scale bar = 50 μm.

As shown in Figure 4A-L, the numbers of blood and lymphatic microvessels were determined by a double immunohistochemical staining with the blood vessel endothelial cell marker CD34 and lymphatic vessel endothelial marker Lyve-1. Numbers of blood microvessels with CD34^+^/Lyve-1^- ^and lymphatic microvessels with CD34^-^/Lyve-1^+ ^were counted on the merged images (Figure 4C, F, I, L). The results of the analysis showed that the number of blood microvessels was significantly lower in the pEndo group, and the number of lymphatic microvessels was significantly decreased in the pesVEGFR-2 and pEndo groups, as compared to the corresponding pVec group (Figure 3C).

### Dilated lymphatic vessels with cancer cell invasion

**Figure 5:**
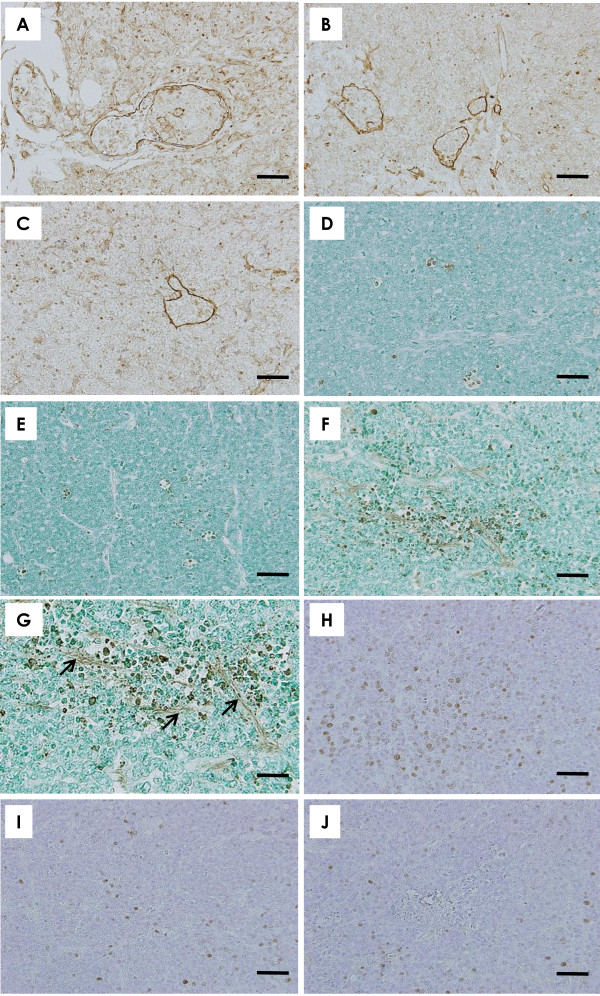
**Dilated lymphatic vessels with cancer cell invasion, apoptosis, and cell proliferation in mammary carcinomas**. Podoplanin-positive lymphatic microvessels of a tumor in a control mouse were often dilated and filled with tumor cells (**A**). The pesVEGFR-2 (**B**) and pEndo (**C**) groups showed a reduction in the numbers of dilated lymphatic microvessels containing intralumenal tumor cells (**A-C**, scale bar = 50 μm). Whereas some TUNEL-positive cells are seen in the tumor of a control mouse (**D**) and a mouse treated with pesVEGFR-2 (**E**), many more TUNEL-positive cells are observed in the tumor of a mouse treated with pEndo (**F**) (**D-F**, scale bar = 50 μm). Higher magnification in **F **showed many TUNEL-positive cells are observed in along with brownish necrotic vessels (**G**, arrows) (scale bar = 25 μm). Apoptosis of the tumor cells may be due to injury of the blood vessels (blockage of oxygen supply and nutrition to tumor cells). The number of BrdU-labeled cells tended to be lower in the pesVEGFR-2 (**I**) and pEndo (**J**) groups than in the pVec group (**H**) (**H-J**, scale bar = 50 μm). **A-C**, podoplanin immunohistochemistry; **D-G**, TUNEL stain; **H-J**, BrdU immunohistochemistry.

The lymphatic microvessels in the mammary tumors were also stained for another lymphatic endothelial marker podoplanin, as demonstrated in Figure 5A-C. Frequently, tumor cells within the lumina of dilated lymphatic vessels in the tumors were observed in both control (Figure 5A) and treated animals (Figure 5B, C). As shown in Figure 3D, the number of lymphatic vessels having intralumenal cancer cells was significantly lower in the pesVEGFR-2 and pEndo groups as compared to the pVec group, supporting the suppression of lymph node metastasis in these groups.

### Apoptosis and cell proliferation in mammary tumor cells

Results of quantitative analysis for apoptosis in lesions, as assessed by the TUNEL assay, are shown in Figure 5D-G; the number of TUNEL-positive cells was significantly increased in tumors from the pEndo group (Figure 3E, Figure 5F, G) as compared to levels seen in tumors from control mice of the pVec group (Figure 5D). No apparent differences of apoptosis levels in tumors were observed between the pVec (Figure 5D) and pesVEGFR-2 (Figure 5E) groups. Cell proliferation, assessed by BrdU immunohistochemistry (Figure 5H-J), was significantly reduced in mammary tumor cells treated with pesVEGFR-2 or pEndo than in those observed in corresponding control tumors (Figure 3F).

## Discussion

In the present study, gene therapy with vectors expressing esVEGFR-2 significantly suppressed the multiplicity of lymph node metastasis and lung metastatic nodules in an immunocompetent metastatic mammary cancer model, whereas pEndo (as a positive control) strongly inhibited overall metastasis. Survival rates tended to be prolonged in the pesVEGFR-2 and pEndo groups, although this tendency was not statistically significant. Tumor volume was significantly reduced in the pesVEGFR-2 and pEndo groups, and this reduction was associated with decreased cell proliferation as assessed by BrdU labeling indices. In addition, the antitumor effects in the pEndo group were significantly stronger than the antitumor effects in the pesVEGFR-2 group. The inhibition of metastasis in these groups may simply be a reflection of the suppressed tumor growth and cell proliferation. However, this therapeutic benefit is apparent because conventional therapies are often insufficient to eradicate metastatic breast cancer. When the diameter of malignant breast tumors reaches 4 cm or larger, the chance of tumor recurrence and/or metastasis increases dramatically [24]. The prolonged survival, reduced tumor volume, and suppression of metastasis after pesVEGFR-2 therapy suggests that esVEGFR-2 may potentially represent a novel therapy for cancer treatment.

Tumor cell dissemination is mediated by a number of mechanisms, including direct invasion into local tissue, lymphatic spread, and hematogenous spread. The most common pathway of initial dissemination is via the afferent ducts of the lymphatics [25]. The lymphatic capillaries present in tissues and tumors provide entrance into the lymphatic system, allowing cancer cell migration to the lymph nodes. VEGF-C expression correlates with lymph node metastasis in a variety of human cancers, including breast neoplasms [8,26]. In many animal models of cancer, VEGF-C enhances tumor lymphangiogenesis, the metastatic spread of tumor cells to lymph nodes and, in some cases, distant organ metastasis [27]. Downregulation of VEGF-C with siRNA reduces lymph node metastasis in murine mammary cancer models [20,28]. In addition, VEGFR-3, the VEGF-C receptor, is predominantly expressed on lymphatic endothelial cells [29], and VEGF-C-dependent activation of VEGFR-3 stimulates the growth of lymphatic endothelial cells and lymphatics [30]. Blockade of VEGFR-3 signaling by sVEGFR-3 or blocking antibody inhibits lymph node metastasis in experimental animal cancer models and is associated with a reduction in lymphangiogenesis but not angiogenesis of tumors [2-4]. In contrast, Laakkonen *et al*. reported that VEGFR-3 blocking antibody therapy significantly suppresses both angiogenesis and lymphangiogenesis [31]. In addition, Burton *et al*. reported that sVEGFR-3 significantly inhibits lymphangiogenesis and slightly inhibits tumor blood vasculature. They speculated that the inhibition of tumor blood vasculature could likely be responsible for the delay in tumor growth *in vivo *[5].

We recently demonstrated that naturally occurring esVEGFR-2 is a VEGF-C antagonist that selectively inhibits lymphangiogenesis and is associated with normal alymphatic cornea [7]. In fact, the present study shows that the multiplicity of lymph node metastasis and lung metastatic nodules was significantly reduced in the pesVEGFR-2 group and associated with a decreased number of lymphatic vessels but not blood vessels in mammary carcinomas. However, as shown in Table 1, pesVEGFR-2 did not decrease the number of unilateral or bilateral metastasis in the lungs, ovaries, kidneys and adrenals, which are types of hematogenous metastasis. Thus, treatment with pesVEGFR-2 that primarily inhibits lymphangiogenesis may be ineffective in this experimental setting. But, since pesVEGFR-2 significantly decreased the number of metastatic nodules in the lungs, some possibilities are raised. An initial pathway of lung metastasis may also be through the lymphatic pathway (thoracic duct); cancer cells then influx into the left subclavian vein, pass through the right ventricle of the heart and pulmonary artery, and then settle and grow in the lung tissue. In addition, cancer cells metastasize to lymph nodes and invade into blood microvessels within the lymph node and then hematogenously spread to the lungs. If so, the number of cancer cells that metastasize to the lungs may be decreased. Alternatively, the secreted esVEGFR-2 in blood may inhibit the survival of cancer cells circulating in the blood, or it may inhibit the settlement of cancer cells in the lungs. Further investigation is necessary to explore these possibilities. In addition, we observed a significant decrease in the number of lymphatic vessels with tumor cells in their lumina in the pesVEGFR-2 and pEndo groups as compared to the pVec control group. This finding indicates an inhibitory effect on migration into tumor lymphatic vessels that supports a significant reduction in lymph node metastasis in these groups. In addition, the number of CD8^+ ^T cells and dendritic cells is significantly increased in inoculated murine mammary tumor cells stably transfected with VEGF-C siRNA, suggesting that VEGF-C modulates the immune response [28]. Therefore, the immune response may participate in the antimetastatic potential of pesVEGFR-2 in the immunocompetent mammary cancer model in the present experiment.

Previous studies have found that the systemic administration of the anti-VEGFR-3 blocking antibody inhibits lymph node metastasis and reduces lymphatic vessel density in orthotopic lung [2] or gastric tumors [3] in nude mice. However, there were no changes in angiogenesis or tumor weight. The results of the present study raise the question of why esVEGFR-2 suppresses tumor growth without suppressing tumor angiogenesis. VEGF-C induces tumor growth in orthotopic prostate tumors [32] or gastric carcinomas [33] in nude mice. Indeed, in the present study, esVEGFR-2 decreased cell proliferation as determined by BrdU-labeling indices. Since esVEGFR-2 is an antagonist of VEGF-C, it is possible that pesVEGFR-2 could inhibit tumor growth as well, as indicated by the present study. On a related note, sVEGFR-3 can not only bind VEGFR-3 but also acts as a trap for VEGF-C, which blocks VEGFR-3 signaling [2,5].

Endostatin is a 20-kDa C-terminal fragment of collagen XVIII that inhibits endothelial cell proliferation and tumor angiogenesis by several mechanisms. These mechanisms include: blocking the binding of VEGF_121 _and VEGF_165 _to the KDR/Flk-1 receptor, which mediates endothelial cell motility and proliferation; blocking phosphorylation of the tyrosine receptor; and blocking activation of the intracellular signaling kinases ERK, p38 MAPK, and p125 FAK [34,35]. Gene therapy with endostatin causes significant tumor growth arrest in various cancers in laboratory animals [36,37]. We previously showed that gene therapy with endostatin suppresses tumor growth and metastasis (lymph nodes and lungs) and is associated with the inhibition of blood vessels and lymphatic vessels in the mouse mammary cancer model [15], which is consistent with the results of the present study. Brideau *et al*. reported that J4 transgenic mice overexpressing endostatin (driven by the keratin K14 promoter) in epidermal basal cells exhibited inhibited angiogenesis and lymphangiogenesis in skin tumors induced by a carcinogen followed by a tumor promoter agent [16]. The skin tumors in the J4 transgenic mice were less aggressive than tumors in wild-type mice [16]. Thus, endostatin inhibits both blood vessels and lymphatic vessels. Endostatin is a naturally occurring molecule like pesVEGFR-2, not a recombinant protein; hence, we selected endostatin as a positive control. In the present study, pEndo strongly suppressed overall metastasis (lymphatic and hematogenous metastasis) and was associated with decreased angiogenesis and lymphangiogenesis in tumors. A possible reason why metastasis in the pesVEGFR-2 group was not strong as compared to the pEndo group is that pesVEGFR-2 inhibited tumor lymphangiogenesis but not angiogenesis.

## Conclusions

We have demonstrated that gene therapy with alternative splicing variant esVEGFR-2 (a new specific inhibitor of lymphangiogenesis [7]) significantly suppresses tumor growth and lymph node metastasis in a mouse mammary cancer model. In most types of cancer, the first site of metastasis is lymph nodes, and the extent of lymph node involvement is a major criterion for evaluating patient prognosis. Therefore, we believe the antimetastatic activity of esVEGFR-2 may have great clinical significance for the treatment of metastatic human breast cancer.

## Abbreviations

esVEGFR: endogenous soluble vascular endothelial growth factor receptor; H&E: hematoxylin and eosin; LSAB: labeled streptavidin-biotin; MMTV: mouse mammary tumor virus; pEndo: endostatin expression vector; TUNEL: terminal deoxynucleotidyl transferase-mediated dUTP-FITC nick end-labeling; VEGF: vascular endothelial growth factor;

## Competing interests

The authors declare that they have no competing interests.

## Authors' contributions

MS and ES carried out all experiments. RA performed esVEGFR-2 vector construction. Transplantation was performed by JM. Immunofluorescence staining was conducted by MS in consultation with YI. MS wrote the manuscript in consultation with RA and JA. JA edited the manuscript and assisted in experimental design. YO is a head in the department. All authors have read and approved the final manuscript to be submitted.

## Pre-publication history

The pre-publication history for this paper can be accessed here:

http://www.biomedcentral.com/1741-7015/8/69/prepub
